# Development of a strictly regulated xylose-induced expression system in *Streptomyces*

**DOI:** 10.1186/s12934-018-0991-y

**Published:** 2018-09-21

**Authors:** Yuji Noguchi, Norimasa Kashiwagi, Atsuko Uzura, Chiaki Ogino, Akihiko Kondo, Haruo Ikeda, Masahiro Sota

**Affiliations:** 1 Nagase R&D Center, Nagase & Co., Ltd., 2-2-3 Murotani, Nishi-ku, Kobe, Hyogo 651-2241 Japan; 2 Graduate School of Science, Technology and Innovation, Kobe University, 1-1 Rokkodai-cho, Nada-ku, Kobe, Hyogo 657-8501 Japan; 3 Department of Chemical Science and Engineering, Graduate School of Engineering, Kobe University, 1-1 Rokkodai-cho, Nada-ku, Kobe, Hyogo 657-8501 Japan; 4 RIKEN Center for Sustainable Resource Science, 1-7-22 Suehiro-cho, Tsurumi-ku, Yokohama, Kanagawa 230-0045 Japan; 5 Laboratory of Microbial Engineering, Kitasato Institute for Life Sciences, Kitasato University, 1-15-1 Kitasato, Minami-ku, Sagamihara, Kanagawa 252-0373 Japan

**Keywords:** Xylose, Xylose isomerase promoter, *Streptomyces avermitilis*, *Streptomyces lividans*, Heterologous protein production

## Abstract

**Background:**

Genetic tools including constitutive and inducible promoters have been developed over the last few decades for strain engineering in *Streptomyces*. Inducible promoters are useful for controlling gene expression, however only a limited number are applicable to *Streptomyces*. The aim of this study is to develop a controllable protein expression system based on an inducible promoter using sugar inducer, which has not yet been widely applied in *Streptomyces*.

**Results:**

To determine a candidate promoter, inducible protein expression was first examined in *Streptomyces avermitilis* MA-4680 using various carbon sources. Xylose isomerase (*xylA*) promoter derived from xylose (*xyl*) operon was selected due to strong expression of xylose isomerase (XylA) in the presence of d-xylose. Next, a xylose-inducible protein expression system was constructed by investigating heterologous protein expression (chitobiase as a model protein) driven by the *xylA* promoter in *Streptomyces lividans*. Chitobiase activity was detected at high levels in *S. lividans* strain harboring an expression vector with *xylA* promoter (pXC), under both xylose-induced and non-induced conditions. Thus, *S. avermitilis xylR* gene, which encodes a putative repressor of *xyl* operon, was introduced into constructed vectors in order to control protein expression by d-xylose. Among strains constructed in the study, XCPR strain harboring pXCPR vector exhibited strict regulation of protein expression. Chitobiase activity in the XCPR strain was observed to be 24 times higher under xylose-induced conditions than that under non-induced conditions.

**Conclusion:**

In this study, a strictly regulated protein expression system was developed based on a xylose-induced system. As far as we could ascertain, this is the first report of engineered inducible protein expression in *Streptomyces* by means of a xylose-induced system. This system might be applicable for controllable expression of toxic products or in the field of synthetic biology using *Streptomyces* strains.

**Electronic supplementary material:**

The online version of this article (10.1186/s12934-018-0991-y) contains supplementary material, which is available to authorized users.

## Background

*Streptomyces* strains are aerobic, gram-positive, mycelia-forming soil bacteria with high G+C content DNA. These strains have produced many useful compounds such as secondary metabolites [1, 2]. They are also used as industrial strains to produce various products such as commercial antibiotics and antifungals for therapeutic, environmental, and agricultural applications. *Streptomyces* strains also enable the secretion of various hydrolytic enzymes into culture medium; hence, they are useful for native and heterologous protein production as a secretion from culture medium [3, 4]. This secretion property might also be employed to eliminate endotoxin contamination to simplify purification processes, and to fold proteins precisely [5, 6].

Genetic tools have been developed for *Streptomyces*, and these tools have been employed for strain engineering and synthetic biology applications [7]. Promoters are one key factor for protein expression, and development of inducible and constitutive promoters has been reported over the last few decades for *Streptomyces* [3, 8]. Inducible promoters are useful for controlling gene expression in basic and applied studies. For example, the expression level of interesting genes can be changed at certain stages in order to investigate function, or for application in synthetic biology. Inducible promoters can also control the concentration of toxic products to limit the impact on cell growth. As yet, however, only a limited number of inducible promoters, such as P_*tipA*_ and P_*nitA*_, have been developed for *Streptomyces* [9–11].

Inducible promoters by sugar inducers allow for the strict regulation of gene expression in target proteins using inexpensive input [12], and the promoters have been widely developed for protein expression in various species, including xylose-regulation systems for protein expression that include P_*xyl*_ (*Bacillus subtilis* [13], *Clostridium perfringens* [14], *Brevibacillus choshinensis* [15]), and P_*xylT*_ (*Lactococcus lactis* [12]). Other systems using sugar inducers, such as arabinose and rhamnose, have been developed for *Escherichia coli* [16, 17]. *Streptomyces* strains have various specific permeases and are able to metabolize various carbon sources [18], however engineered inducible promoters by sugar inducers have not yet been widely applied for protein expression in *Streptomyces*.

The aim of this study is to develop an engineered inducible protein expression system for *Streptomyces* based on a sugar inducer. Xylose isomerase (*xylA*) promoter and xylose (*xyl*) operon were chosen in an initial attempt to investigate induced protein expression in a *Streptomyces* strain using various carbon sources. *xylA* promoter is a useful candidate for protein expression because actinomycetes strains are intrinsically good XylA protein producers under the control of *xylA* promoter [19]. x*ylA* promoter from *Actinoplanes missouriensis* provides a system for overexpressing Cel6 protein in *Streptomyces lividans*, which is not inducible [20]*. xylA* promoter was also employed for genetic modification, such as efficient expression of *cre* gene for deletion of the 1.5 Mb region in *Streptomyces avermitilis* chromosome [21]. In the present study, an inducible protein expression system was designed under the control of *xylA* promoter with the components of XylR and d-xylose. Strictly regulated protein expression was finally achieved by d-xylose via constructed vector.

## Results

### *Streptomyces avermitilis* MA-4680 protein expression profiles for different carbon sources

**Fig. 1:**
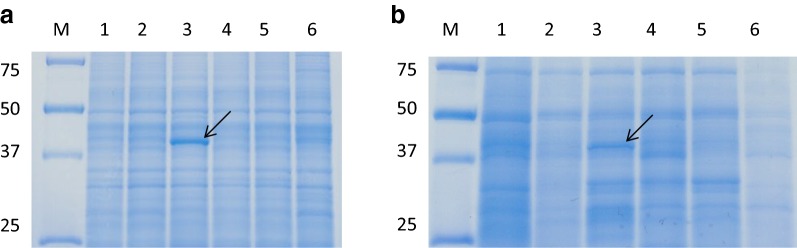
SDS-PAGE analysis of *S. avermitilis* protein expression in **a** cell extracts and **b** culture supernatants: 2.0% 1. Glycerol, 2. d-glucose, 3. d-xylose, 4. d-galactose, 5. d-fructose, and 6. no sugar. M: molecular weight marker. Arrows indicate Xylose isomerase (XylA) protein

Inducible protein expression was first investigated in *Streptomyces avermitilis* MA-4680 using different carbon sources (glycerol, d-glucose, d-xylose, d-galactose, and d-fructose). Protein expression profiles of cell extracts and culture supernatants are shown for each carbon source (2.0%) in Fig. 1 (whole gels shown in Additional file 1: Figure S1). One protein was clearly detected above 37 kDa in the presence of d-xylose from both the cell extract and the culture supernatant (Fig. 1, arrows). On the other hand, this protein band was not detected in the presence of other carbon sources. The detected protein was identified as putative xylose isomerase (XylA, SAV_7182) by MALDI-TOF–MS analysis after the purification of cell extracts and culture supernatants. XylA expression was also detected for this strain in the presence of 1.0% d-xylose (Additional file 2: Figure S2). These results suggest that the expression of the *xylA* gene was induced in *S. avermitilis* by the addition of d-xylose.

### Genetic organization of xylose operon in *S. avermitilis* and mechanism of d-xylose metabolism

**Fig. 2:**
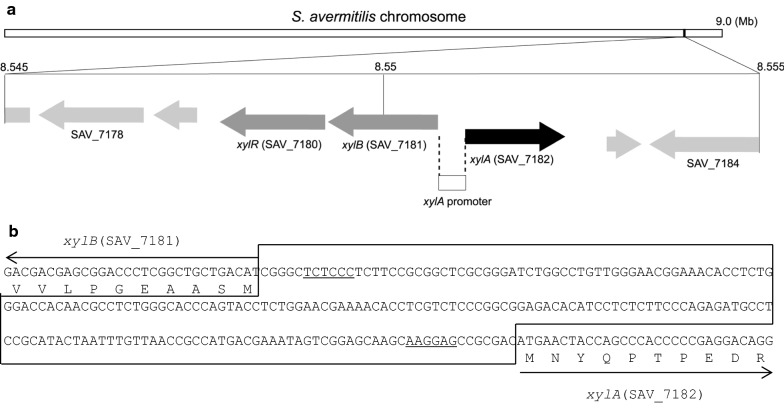
**a** Gene organization in the intergenic region containing the *xyl* operon of *S. avermitilis* MA-4680 from the complete genome sequence. **b** The nucleotide sequence of the intergenic promoter region of the divergently transcribled *xylA* and *xylB* genes of *S. avermitilis*. The putative ribosome binding sites from *S. rubiginosus* are shown as underlined sequences [24]

**Fig. 3:**
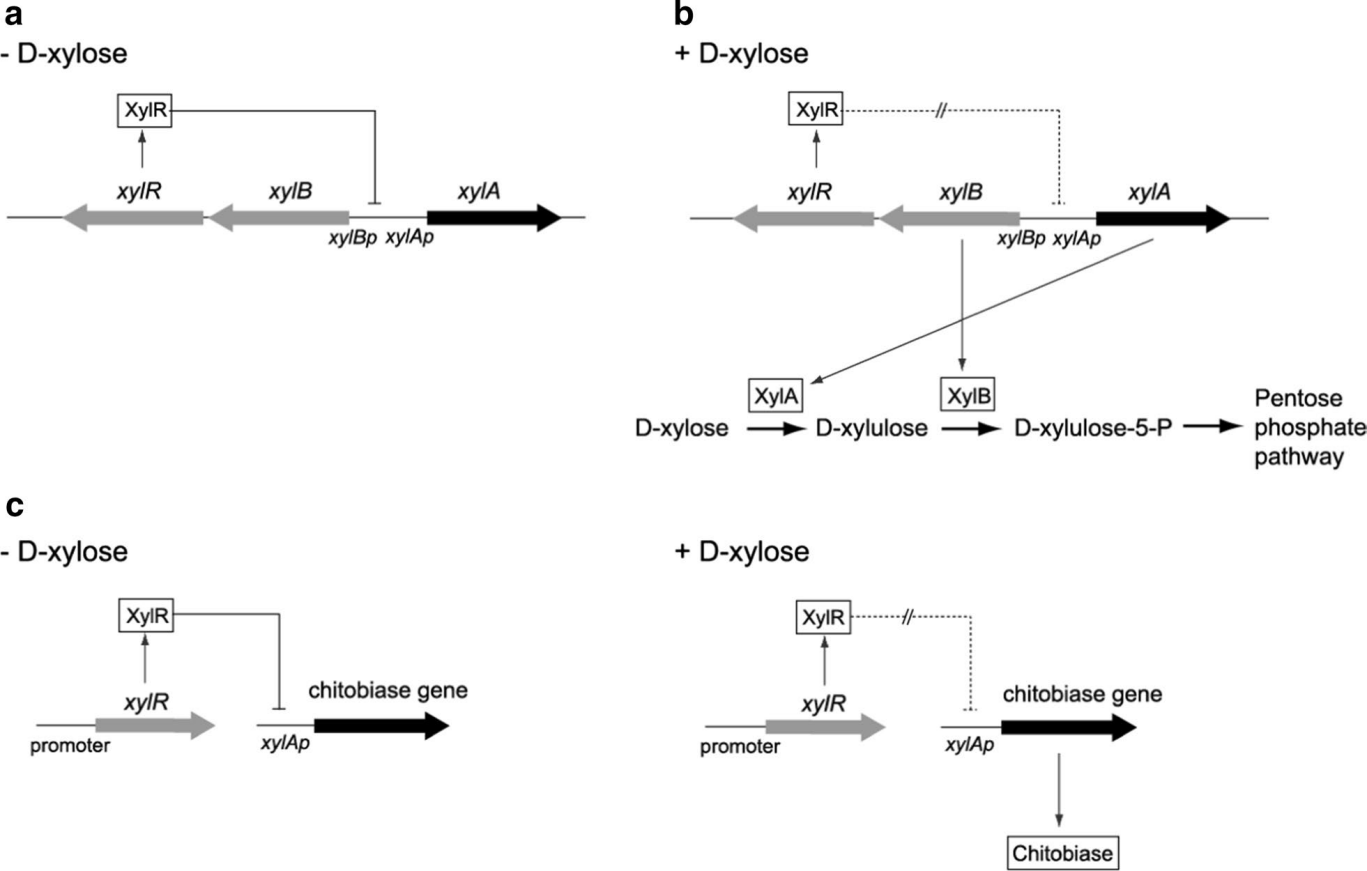
Genetic organization of *Streptomyces xyl* operon and d-Xylose metabolism. Schematic representation of putative gene regulation **a** in the absence of d-xylose and **b** in the presence of d-xylose. **c** Conceptual diagram of projected study

As mentioned above, *S. avermitilis* XylA was induced in the presence of d-xylose. The *xylA* gene is located in xylose (*xyl*) operon, which consists of *xylA* (SAV_7182; xylose isomerase)*, xylB* (SAV_7181; xylulose kinase), and *xylR* (SAV_7180; xylose operon regulator) genes from the complete genome sequence of *S. avermitilis* MA-4680 [22] (Fig. 2). The *xyl* operon is related to d-xylose metabolism, which involves the transport of d-xylose, the isomerization of d-xylose to d-xylulose (mediated by XylA), and the phosphorylation of d-xylulose to d-xylulose-5-phosphate (mediated by XylB) (Fig. 3a, b). XylR acts as a repressor for *xyl* operon in *S. lividans* TK24, as reported in a deletion study of the *xylR* gene [23]. Genes driven by *xyl* promoters are induced in the presence of d-xylose [23, 24] (Fig. 3a, b). *xylA* promoter and *xyl* operon were selected in order to develop a xylose-dependent protein expression system in *Streptomyces* (Fig. 3c).

### Chitobiase expression of the xylAp expression system in *Streptomyces* XC strain

**Fig. 4:**
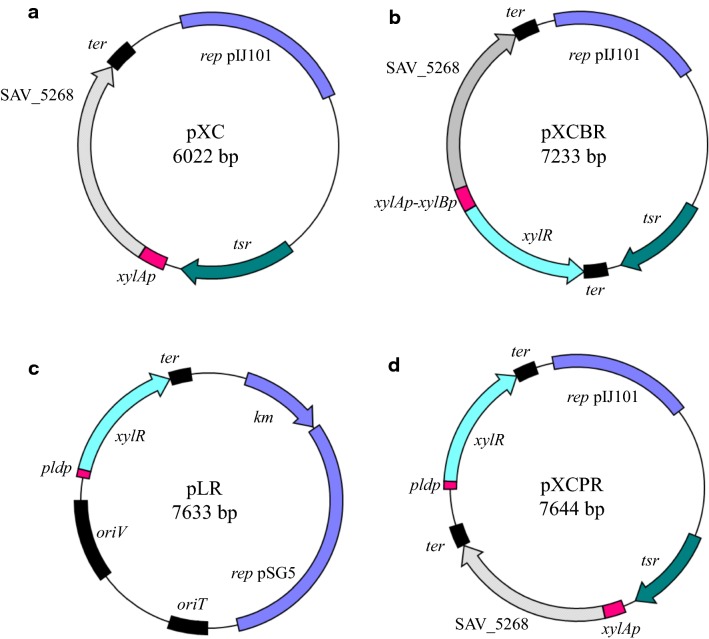
Schematic representation of xylose-induced expression vectors pXC, pXCBR, pLR, and pXCPR. *oriT*, origin of transfer; *oriV*, replication origin of *E. coli* from pK18mob (ATCC^®^ 87095™); *rep* pIJ101 and *rep* pSG5, replicon of *Streptomyces* from pIJ101 [25] and pGM160 [36]; *tsr*, thiostrepton resistance gene; *km*, kanamycin resistance gene

pXC vector was first constructed as a high-copy vector for protein expression under *S. avermitilis xylA* promoter without *xylR* gene in the same vector (Fig. 4a). The *nagZ4* gene (SAV_5268), which encodes chitobiase in *S. avermitilis*, was employed as a reporter in order to investigate the system using the *xylA* promoter. The protein encoded by this gene has a putative signal peptide sequence, and the secretion of the protein was previously confirmed in culture supernatant (data not shown). This vector was transformed into *S. lividans* 1326 (named XC strain in Table 1).

**Table 1:** Plasmids and strains used in this study

Plasmid or strain	Relevant feature
Plasmid	
pIJ350	Versatile vector for protein expression; thiostrepton resistance marker
pUC19	Vector for cloning genes
pGM160	A shuttle vector containing pSG5 *ori*
pK18mob	Cloning vector; *oriT, oriV*; kanamycin resistance marker
pXC	Vector for expression of chitobiase gene (SAV_5268) under the control of *xylA* promoter; thiostrepton resistance marker
pXCBR	Vector for expression of chitobiase gene (SAV_5268) under the control of *xylA* promoter and *xylR* gene under the control of *xylB* promoter; thiostrepton resistance marker
pLR	Vector containing pSG5 *ori* for expression of *xylR* gene under the control of *pld* promoter; kanamycin resistance marker
pXCPR	Vector for expression of chitobiase gene (SAV_5268) under the control of *xylA* promoter and *xylR* gene under the control of *pld* promoter; thiostrepton resistance marker
Strain	
*Escherichia coli* strain JM109	*recA1 endA1 gyrA96 thi*-*1 hsdR17* (rK− mK+) e14− (*mcrA*−) *supE44 relA1* Δ(*lacproAB*)/F’[*traD36 proAB *+ *lacI* q *lacZ*ΔM15]
*S. lividans* 1326	WT strain
XC	*Streptomyces lividans* strain harboring pXC vector
XCBR	*Streptomyces lividans* strain harboring pXCBR vector
XCr	*Streptomyces lividans* strain harboring pXC and pLR vectors
XCPR	*Streptomyces lividans* strain harboring pXCPR vector

**Fig. 5:**
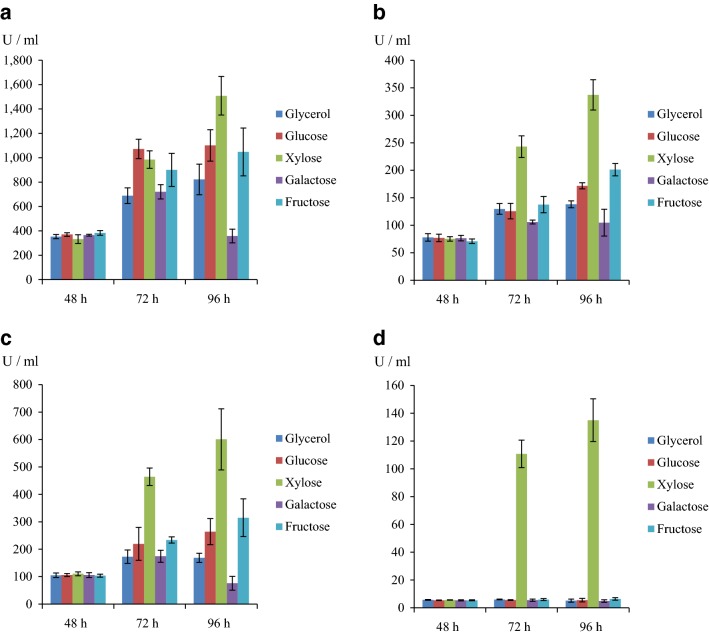
Chitobiase activity of **a** *S. lividans* XC, **b** *S. lividans* XCBR, **c** *S. lividans* XCr, and **d** *S. lividans* XCPR after 48, 72, and 96 h cultivation. After 48 h, carbon sources (glycerol, d-glucose, d-xylose, d-galactose, and d-fructose) were added to a final concentration of 1.0%, and cultivation was continued for 24 and 48 h (72 and 96 h total)

Xylose-induced chitobiase expression was investigated using the *S. lividans* XC strain. The strain was incubated for 48 h, at which time carbon sources (glycerol, d-glucose, d-xylose, d-galactose and d-fructose) were added to final concentration of 1.0%, and incubation continued for an additional 48 h (96 h total cultivation). This strain exhibited remarkable chitobiase activity at 48 h before addition of the carbon sources. After addition of carbon sources (glycerol, d-glucose, d-xylose, d-galactose and d-fructose) and further incubation to 96 h, activity was found to have increased 2.3, 3.0, 4.5, 1.0, and 2.7 times, respectively (Fig. 5a). The highest activity was detected in the presence of d-xylose at 96 h, and higher activity was detected with d-glucose compared to d-xylose at 72 h. The XC strain expressed chitobiase in the presence of all carbon sources. There is no evidence that *xylA* promoter is controlled by d-xylose, and *xylA* promoter appears to be constitutively expressed.

### Chitobiase expression of the xylAp-XylR expression system in the XCBR strain

In order to develop a controllable expression system, pXCBR vector was constructed based on the *S. avermitilis* xylose (*xyl*) operon (Fig. 4b). pXCBR is a high-copy vector for chitobiase gene expression driven by the *xylA* promoter, and *S. avermitilis xylR* gene was introduced in the same vector. *xylR* gene is expressed under the control of *xylB* promoter in a common intergenic region between *xylA* (SAV_7182) and *xylB* (SAV_7181) genes derived from *S. avermitilis* chromosome. The expression vector was transformed into *S. lividans* 1326 (named XCBR strain in Table 1).

Chitobiase activity was investigated in the XCBR strain. Incubation and addition of carbon sources were carried out as described above. This strain exhibited a certain amount of chitobiase activity before addition of the carbon sources (Fig. 5b). With the addition of glycerol, d-glucose, d-xylose, d-galactose, and d-fructose, activity at 96 h was found to have increased 1.8, 2.2, 4.5, 1.4, and 2.8 times, respectively. d-xylose induced slightly higher chitobiase activity than other carbon sources at 72 and 96 h. These results suggest that the *xylA* promoter was slightly regulated by XylR expression in the intergenic region, but such regulation was insufficient when using the region of divergent promoters.

### Chitobiase expression of the xylAp-XylR expression system in the XCr and XCPR strains

Different sets of basic vector and promoter were employed to construct pLR and pXCPR (Fig. 4c, d). The pLR vector is based on the medium copy number plasmid pSG5, while the pXCPR vector is based on the high copy number plasmid pIJ101 [25]. *Streptomyces cinnamoneum* phospholipase D (PLD) promoter was selected to control *xylR* gene expression in pLR and pXCPR vectors [26]. pXC (vector for expression of chitobiase gene, Fig. 4a) and pLR vectors were transformed into *S. lividans* 1326 (named XCr strain in Table 1). pXCPR vector was transformed into *S. lividans* 1326 (named XCPR strain in Table 1).

XCr and XCPR strains were investigated for chitobiase expression using the various carbon sources as described above. The XCr strain exhibited moderate chitobiase activity at 48 h. The addition of 1.0% glycerol, d-glucose, d-xylose, d-galactose, and d-fructose increased activity 1.6, 2.5, 5.4, 0.7, and 3.0 times at 96 h, respectively (Fig. 5c), suggesting that induction is weak in the XCr strain. The XCPR strain, on the other hand, exhibited inducible expression of chitobiase (Fig. 5d). Chitobiase activity was low in this strain before addition of the carbon sources. The activity was 24-fold higher after addition of d-xylose at 96 h, while the activity remained low after the addition of other carbon sources. These results indicate that the XCPR strain strictly regulates the *xylA* promoter with d-xylose. Chitobiase activity was also investigated in the XCPR strain with the different d-xylose concentrations from 0.0 to 2.0% at 48, 72, and 96 h (Additional file 3: Figure S3). The difference in activity between 1.0 and 2.0% was found to be slight, and growth was barely affected with different d-xylose concentrations from 0.0 to 2.0%.

## Discussion

An inducible promoter would be useful in *Streptomyces* for such applications as controllable expression of heterologous protein, or as a genetic tool for expressing interesting genes at certain stages [7, 8, 10]. The aim of this study is to develop a controllable gene expression system for *Streptomyces* strains based on inducible promoter related with carbon sources. A candidate inducible promoter was first selected by analyzing induced proteins with different carbon sources, and an inducible protein expression system was constructed driven by the candidate promoter.

Protein induction was observed with different carbon sources in *S. avermitilis* MA-4680. *S. avermitilis* is particularly well-known for avermectin production [27], and the complete genome sequence of this strain has already been reported [22]. In the analysis of induced proteins using various carbon sources as inducer, XylA protein was found to be well expressed with d-xylose in the cell extract and the culture supernatant (Fig. 1). Extracellular secretion of XylA in some *Streptomyces* species has been reported [19], but this is fairly uncommon because it does not contain a putative secreted signal sequence. The XylA detected in culture supernatants might have been released from the cell due to cell wall permeability, or by partial lysis of the cells [19].

The *xylA* gene exists in the *xyl* operon from the complete genome sequence of *S. avermitilis* MA-4680 (Fig. 2) [22]. The *S. avermitilis xyl* operon consists of *xylA* (SAV_7182), *xylB* (SAV_7181), and *xylR* (SAV_7180) genes. In comparison, *S. coelicolor* A3 (2) [28] and *S. lividans* 1326 [29] show the same gene organization such as *xylA* (SCO1169 or SLI_1446), *xylB* (SCO1170 or SLI_1447), and *xylR* (SCO1171 or SLI_1448) genes (Additional file 4: Figure S4). On the other hand, the organization of the *xylA*, *xylB* and *xylR* locus in *S. griseus* (NBRC 13350) [30] is a little different, such as *xylR* (SGR_1069), *xylA* (SGR_1070), and *xylB* (SGR_1071) genes (Additional file 4: Figure S4). *xyl* gene regulatory mechanism by regulatory protein differs among species. The regulator functions as an activator in *E. coli*, but as a repressor in *Bacillus* [31–33]. Regulatory protein (XylR) acts as a repressor for *xyl* operon in *Streptomyces* TK24, as reported in a deletion study of the *xylR* gene [23]. In the present study, the *xylA* promoter and *xyl* operon were selected in order to develop a xylose-dependent protein expression system in *Streptomyces* (Fig. 3c).

A set of protein expression vectors was constructed based on *S. avermitilis xylA* promoter and the *xyl* operon (Fig. 4). Extracellular chitobiase activity was investigated in *S. lividans* strain harboring these vectors. *S. lividans* is known as an attractive host for heterologous protein production because it has a low level of endogenous proteolytic activity, and plasmid-based expression systems have been established [4, 5]. Expression of extracellular chitobiase from *S. avermitilis* was examined as a model. *S. lividans* also contains a putative chitobiase gene in its chromosome (SLI_3133), however *S. lividans* wild-type strain tested under the same conditions as in the present study displayed no intrinsic chitobiase activity.

In the strain harboring pXC vector without *S. avermitilis xylR* gene in the same vector, activity was detected under both xylose-induced and non-induced conditions (Figs. 4a, 5a), indicating that the XC strain is not capable of controlling protein expression with d-xylose. *xylR* gene functioning as a repressor also exists in the *S. lividans* chromosome, but intrinsic XylR might be insufficient for regulation of the *S. avermitilis xylA* promoter, presumably because of the high-copy number of the *xylA* promoter in the vector as opposed to the expression level of *xylR* gene in the chromosome.

From these results, vectors were constructed containing the *S. avermitilis* XylR component in order to repress *xylA* promoter activity in the absence of d-xylose. In the XCBR vector, the common intergenic region between *xylA* and *xylBR* genes was employed for the expression of *S. avermitilis xylR* gene (Figs. 2 and 4b). Common intergenic regions exist in several bacteria species, such as between *xylR* and *xylAB* genes in *Bacillus subtilis* and between *xylR* and *xylBA* genes in *Clostridium*, although gene organization is different in *Streptomyces* [13, 14]. Divergent promoters in these regions are easily employed to control *xylR* gene for vector construction of an inducible target protein expression in these strains [13, 14]. In the present study, the intergenic region of *Streptomyces xyl* operon was employed to express the *xylR* gene by placing the gene under the control of *xylB* promoter (Fig. 4b). Although protein induction occurred using the constructed vector, the induction ratio was weak in the *Streptomyces* strain (XCBR strain in Fig. 5b).

From the result of protein expression using the XCBR strain, different sets of promoter and vector were employed for suitable expression of the *S. avermitilis xylR* gene to optimize xylose-induced expression. *S. cinnamoneum* phospholipase D (PLD) promoter was employed for expression of the XylR regulatory protein. The promoter is constitutive, and examples have been reported to express heterologous genes under the control of the promoter in *S. lividans* [3, 34]. Moreover, two different vectors were employed. The pLR vector (Fig. 4c) is based on a medium copy number plasmid, and the pXCPR vector (Fig. 4d) is based on a high copy number plasmid. Xylose-induced expression was investigated using XCr strain (harboring pXC and pLR vectors; Fig. 4a, c), and XCPR strain (harboring pXCPR vector; Fig. 4d). Different protein expression behaviors were observed between these two strains. The XCr strain showed regulation of chitobiase expression at low levels (Fig. 5c), while the XCPR strain exhibited strictly regulated expression at 72 and 96 h (Fig. 5d). XylR expression from pLR (medium copy number, Fig. 4c) might be insufficient to regulate the *xylA* promoter in the XCr strain, but XylR expression was sufficient using pXCPR (high copy number, Fig. 4d) in the XCPR strain. As seen in the XC strain, intrinsic XylR may be insufficient for regulation of this system (Fig. 5a). Results show that strictly regulated xylose-induced expression is achieved by the combination of *xyl* operon components (*xylA* promoter and XylR) for d-xylose metabolism in *Streptomyces*.

The engineering of a strictly regulated xylose-induced system has only just begun, and further study is needed for practical applications using d-xylose, such as controlling the expression of toxic recombinant proteins, or gene expression in the field of synthetic biology. Moreover, the d-xylose induced system might be employed using mixed sugars such as d-glucose. Gene expression was induced in the presence of 1% (w/v) d-xylose and d-glucose in *S. rubiginosus*, although the expression was lower than in the presence of d-xylose alone [24]. The constructed system in the present study may be applicable in such a case.

## Conclusions

In this study, strictly regulated protein expression was achieved, based on a xylose-induced system. First, the induction of XylA in a model *Streptomyces* strain was found to be greater with d-xylose than with other carbon sources. Next, xylose-induced protein expression system was developed using constructed vectors containing *xylA* promoter and XylR components. As far as we could ascertain, this is the first report of engineered inducible protein expression in *Streptomyces* by means of a xylose-induced system. This system might be applicable with *Streptomyces* for controllable expression of toxic products or in the field of synthetic biology.

## Methods

### Bacterial strains in this study

*Streptomyces avermitilis* MA-4680 (NBRC 14893) and *S. lividans* 1326 (NBRC 15675) were purchased from National Institute of Technology and Evaluation (NITE, Chiba, Japan). *Escherichia coli* JM109 (Takara, Shiga, Japan) was used as the host for DNA manipulation (Table 1).

### SDS analysis of *S. avermitilis* MA-4680 induced protein in the presence of different carbons

*Streptomyces avermitilis* MA-4680 was cultured on inorganic salt starch agar plates. A single colony was pre-cultured in TSB medium (17 g/l pancreatic digest of casein, 3 g/l papaic digest of soybean meal, 2.5 g/l glucose, 5 g/l sodium chloride, and 2.5 g/l dipotassium phosphate, Becton, Dickinson and Company, Sparks, MD, USA) for 48 h at 28 °C, then inoculated into modified TSB medium (17 g/l pancreatic digest of casein, 3 g/l papaic digest of soybean meal, 5 g/l sodium chloride, and 2.5 g/l dipotassium phosphate) in a 500 ml baffled flask in the presence of 1.0 or 2.0% various carbon sources (glycerol, d-glucose, d-xylose, d-galactose, and d-fructose) for 96 h at 28 °C. Cells were harvested by centrifugation and re-suspended in 20 mM potassium phosphate buffer (pH 7.0). Suspensions were sonicated and centrifuged. Cell extracts were analyzed by e-PAGEL (15%) (ATTO, Tokyo, Japan). Culture supernatants (200 μl) were desalted with Bio-Spin^®^6 Tris Columns (Bio-Rad, Hercules, CA, USA), then lyophilized. Samples were dissolved by SDS sample buffer (0.125 M Tris–HCl (pH 6.8), 4% (w/v) SDS solution, 20% (w/v) glycerol, 0.01% (w/v) BPB, 0.12 M 2-mercaptoethanol) and loaded onto e-PAGEL (15%). Culture supernatants and cell extracts were concentrated using Amicon^®^ Ultra (Merck Millipore, Co Cork, Ireland). From the samples, fractions including XylA protein were separated via HiTrap Q column (GE Healthcare Bio-Sciences AB, Uppsala, Sweden). After the fractions were concentrated using Amicon^®^ Ultra, XylA protein was purified by SDS-PAGE and analyzed by MALDI-TOF–MS (Bruker Daltonics, Leipzig, Germany).

### Construction of pXC vector

A chitobiase expression vector pXC carrying a xylose isomerase promoter (*xylA*p) was constructed as follows:

Plasmid pXC carries the *Streptomyces avermitilis xylA*p, followed by the coding sequence for SAV_5268 (*nagZ4* gene) and the *Streptomyces cinnamoneum* PLD terminator (*pld*t) on pIJ350 [35]. To construct this plasmid, *xylAp* (the selected region lacks *xylB* RBS from *xylA*-*xylB* divergent promoter region in Fig. 2) and *pldt* fragments were amplified via PCR using primer pairs XIApF-XIpR and PLDtF-PLDtR, respectively (Table 2). These two pairs included 27-bp overlapping sequences containing multi-cloning sites that allowed them to anneal with one another, so that the products worked as primers to amplify a 0.4 kb joined fragment. Plasmid pIJ350 was digested with *Pst*I, the ends of which were blunted by KOD DNA polymerase (Toyobo, Osaka, Japan) and then ligated with a 0.4-kb amplified fragment containing *xylAp* and *pldt*. The resultant vector was subsequently digested with *Aor*51HI and *Eco*T22I and ligated with a *sav5268*-containing fragment, which was amplified by PCR using primers SAV5268F and SAV5268R (Table 2) and then digested with *Eco*T22I. Thus constructed, the plasmid was then named pXC.

**Table 2:** Oligonucleotide primers used in this study

Primer	Sequence
XIApF	5′-CTTCCGCGGCTCGCGGGATCTGGC
XIpR	5′-GCATGCATCTAGAAGCTTGAATTCGCT**AGCGCT**GCGGCTCCTTGCTTGCTCCG
PLDtF	5′-AGCGAATTCAAGCTTCTAGATGCATGCGACGACTGAGCGCCCGGACG
PLDtR	5′-ATTTCCGGTCGGTTCGGGGCCAGCGCAT
PLDpF	5′-ATATATGGTACCGGCTCCCGGGAGCTGATAGC
PLDpR	5′-GCATGCATCTAGAAGCTTGAATTCGCT**AGCGCT**GCATCCTTAAACGAAGTAAC
PLDtR_KpnI	5′-ATATATGGTACCATTTCCGGTCGGTTCGGGGCCAGCGCAT
SAV5268F	5′-ATGAGACAGCACCACAGAACGCC
SAV5268R	5′-ACATGCATGCCTACGCACCGGGCCAGGGAA
XylAp_XylBp_NdeI	5′-ATATATCATATGGCTCTCCCTCTTCCGCGGCTCGCGGGATCTG
XylRF_NdeI	5′-ATATATCATATGACCGCACCGCTGCACGA
PLDtR_PstI	5′-ATATATCTGCAGATTTCCGGTCGGTTCGGGGCCAGCGCAT
XylRF	5′-AATTATAGCGCTATGACCGCACCGCTGCACGA
SG5F_BstBI	5′-TTTTTTCGAACGCGTCGTCGTCGACGGCCT
SG5R_BstBI	5′-TTTTTTCGAAGATCACGAGGTCACTCCGTC
PsXylRF_EcoRI	5′-ATAATTGAATTCGGCTCCCGGGAGCTGATAGC
PtXylRR_HindIII	5′-ATGCATAAGCTTATTTCCGGTCGGTTCGGGGC
XylRR	5′-ATATATATGCATCTACCGGTGCGTCGCCGC

Overlap sequences are underlined

*Aor*51HI restriction sites are shown in bold

### Construction of pXCBR and pXCPR vectors

The inducible chitobiase expression vectors pXCBR and pXCPR, carrying a xylose isomerase promoter (*xylA* promoter) and a *xylR* gene under the control of different promoters, were constructed as follows:

Plasmid pXCBR contains the *xylR* gene under control of *xylB* promoter in a common intergenic region between *xylA* and *xylB* genes. To construct this plasmid, the *xylAp*-*sav5268*-*pldt* fragment was amplified by PCR using the primer pair (XylAp_XylBp_NdeI and PLDtR_KpnI primers) and pXC vector as a template (Table 2). The *xylR*-*pldt* fragment was also amplified by PCR using the primer pair (XylRF_NdeI and PLDtR_PstI primers) and pXCPR vector as a template. The *xylAp*-*sav5268*-*pldt* fragment was digested with *Nde*I and *Kpn*I. The *xylR*-*pldt* fragment was digested with *Nde*I and *Pst*I. These fragments were ligated and inserted into the *Pst*I/*Kpn*I restriction sites of pIJ350, yielding plasmid pXCBR.

Plasmid pXCPR contains the *xylR* gene under control of the *S. cinnamoneum* phospholipase D (PLD) promoter [26]. To construct this plasmid, *pldp* and *pldt* fragments were amplified via PCR using primer pairs PLDpF-PLDpR and PLDtF-PLDtR_KpnI (Table 2). Amplified *pldp* and *pldt* fragments were joined by 27-bp overlapping sequences, and the fragment was amplified via PCR using the primer pair PLDpF-PLDtR_KpnI. The amplified fragment and pUC19 were digested with *Kpn*I and ligated together. The resultant vector was digested with *Aor*51HI and *Eco*T22I and ligated with the *xylR*-containing fragment amplified via PCR using the primer pair XylRF-XylRR and *S. avermitilis* genome as a template. The resultant plasmid was referred to as pPR. The XylR expression cassette *pldp*-*xylR*-*pldt* was obtained from pPR as a *Kpn*I digested fragment. This fragment was inserted into the *Kpn*I site of pXC, yielding the plasmid pXCPR.

### Construction of pLR vector

The pLR vector contains pSG5 origin and was constructed as follows:

pSG5 origin was amplified by PCR using the primer pair (SG5F_BstBI and SG5R_BstBI primers) and pGM160 as a template [36]. The amplified fragment and pK18mob vector were digested with *Bst*BI and ligated together [37]. The resultant vector was subsequently digested with *Eco*RI and *Hin*dIII and was ligated with the XylR expression cassette *pldp*-*xylR*-*pldt*, which was amplified by PCR using PsXylRF_EcoRI and PtXylRR_HindIII primers (Table 2) and digested with *Eco*RI and *Hin*dIII. The constructed plasmid was designated pLR.

### Transformation, culture, and expression of chitobiase in *S. lividans*

*Streptomyces lividans* XC, XCBR, and XCPR were obtained by introducing plasmids pXC, pXCBR, and pXCPR into *S. lividans* 1326 via protoplast transformation [38]. *S. lividans* XCr was obtained by introducing plasmid pLR into *S. lividans* XC. Single colonies of each transformant were pre-cultured in modified TSB medium for 3 days at 28 °C. Seed cultures were then transferred into the TSB medium, and cultivation was performed at 28 °C for 48 h. After 48 h, various carbon sources (glycerol, d-glucose, d-xylose, d-galactose, and d-fructose) were added to the medium to a final concentration of 1.0%, and cultivation was continued at 28 °C for 24 and 48 h (72 and 96 h total). Supernatants were harvested by centrifugation. In order to investigate chitobiase expression in XCPR strain at different d-xylose concentrations, seed cultures were transferred into the modified TSB medium, and cultivation was performed at 28 °C for 48 h. After 48 h, d-xylose was added to final concentrations of 0.0, 0.5, 1.0, and 2.0%. Cultivation was continued at 28 °C for 24 and 48 h (72 and 96 h total).

### Measurement of chitobiase activity

Culture supernatants were measured for chitobiase assay. Chitobiase activity was measured using 4-Nitrophenyl *N*-acetyl-*β*-d-glucosaminide (Sigma-Aldrich, St. Louis, MO, USA) as a substrate. An assay was performed according to an established method [39]. One unit of enzyme activity was defined as the amount of enzyme that releases 1 μmol of 4-nitrophenol per minute. Assays were repeated three times.

## Additional files

**Additional file 1: Figure S1.** SDS-PAGE gels of *S. avermitilis* protein expression profiles for (A) cell extracts and (B) culture supernatants: 2.0% 1. Glycerol, 2. D-glucose, 3. D-xylose, 4. D-galactose, 5. D-fructose, and 6. no sugar. M: molecular weight marker. Arrows indicate Xylose isomerase (XylA) protein.

**Additional file 2: Figure S2.** SDS-PAGE gels of *S. avermitilis* protein expression profiles for (A) cell extracts and (B) culture supernatants: 1.0% 1. Glycerol, 2. D-glucose, 3. D-xylose, 4. D-galactose, 5. D-fructose, and 6. no sugar. M: molecular weight marker. Arrows indicate Xylose isomerase (XylA) protein.

**Additional file 3: Figure S3.** (A) Chitobiase activity and (B) optical density (600 nm) of *S. lividans* XCPR after 48, 72, and 96 h cultivation. After 48 h, D-xylose was added to a final concentration of 0.0, 0.5, 1.0, and 2.0%. Cultivation was continued for 24 and 48 h (72 and 96 h total).

**Additional file 4: Figure S4.** Gene organization in the *xyl* operon of *S. avermitilis* MA-4680, *S. coelicolor* A3 (2), *S. lividans* 1326, and *S. griseus* (NBRC 13350) [22, 28, 29, 30].
