# Supplementary material for: Development of a strictly regulated xylose-induced expression system in Streptomyces
Source: Microb Cell Fact. 2018 Sep 21;17:151. doi: 10.1186/s12934-018-0991-y (PMC6149001; doi:10.1186/s12934-018-0991-y)
Supplement: Supplementary file 3 — Additional file 3: Figure S3. (A) Chitobiase activity and (B) optical density (600 nm) of S. lividans XCPR after 48, 72, and 96 h cultivation. After 48 h, D-xylose was added to a final concentration of 0.0, 0.5, 1.0, and 2.0%. Cultivation was continued for 24 and 48 h (72 and 96 h total). [file 12934_2018_991_MOESM3_ESM.docx]

**Fig. S3**
