# Supplementary material for: Development of a strictly regulated xylose-induced expression system in Streptomyces
Source: Microb Cell Fact. 2018 Sep 21;17:151. doi: 10.1186/s12934-018-0991-y (PMC6149001; doi:10.1186/s12934-018-0991-y)
Supplement: Supplementary file 4 — Additional file 4: Figure S4. Gene organization in the xyl operon of S. avermitilis MA-4680, S. coelicolor A3 (2), S. lividans 1326, and S. griseus (NBRC 13350) [22, 28, 29, 30]. [file 12934_2018_991_MOESM4_ESM.docx]

**Fig. S4**
